# Predictors of early cancer burden in *CDH1* pathogenic variant carriers: a UK single-centre cohort study

**DOI:** 10.1016/j.eclinm.2026.104036

**Published:** 2026-07-02

**Authors:** Lianlian Wu, Hui Jun Lim, Nandini Karthik, Sophie Samra, Maria O'Donovan, J Robert O'Neill, Marc Tischkowitz, Rebecca C. Fitzgerald, Massimiliano Di Pietro

**Affiliations:** a Early Cancer Institute, University of Cambridge, Cambridge, United Kingdom; b School of Clinical Medicine, University of Cambridge, Cambridge, United Kingdom; c Department of Histopathology, Cambridge University Hospitals NHS Foundation Trust, Cambridge, United Kingdom; d Department of Surgery, University of Cambridge, Cambridge, United Kingdom; e Cambridge Oesophago-gastric Centre, Cambridge University Hospitals NHS Foundation Trust, Cambridge, United Kingdom; f Department of Genomic Medicine, National Institute for Health Research, Cambridge Biomedical Research Centre, University of Cambridge, Cambridge, United Kingdom; g Department of Gastroenterology, Digestive Diseases, Cambridge University Hospitals NHS Foundation Trust, Cambridge, United Kingdom

**Keywords:** Hereditary diffuse gastric cancer, Signet ring cell carcinoma, CDH1, Prophylactic gastrectomy, Endoscopic surveillance, Cancer burden

## Abstract

**Background:**

Hereditary diffuse gastric cancer (HDGC) with *CDH1* germline pathogenic variants carries a high lifetime risk of signet ring cell carcinoma (SRCC). There is limited data to inform decision between endoscopic surveillance and prophylactic total gastrectomy (PTG) in patients with early SRCC, which could have indolent behaviour. We hypothesise patients with few early SRCC lesions can be monitored endoscopically. Therefore, we aimed to identify clinical predictors early SRCC burden and evaluate the natural histoary of early cancer lesions in HDGC.

**Methods:**

This is a single centre longitudinal cohort study of CDH1 germline variant carriers recruited at Cambridge University Hospitals (United Kingdom) between 2005 and 2024. We analysed two cohorts: 53 patients who underwent endoscopic evaluation followed by PTG and 93 individuals who received longitudinal surveillance. We excluded patients with advanced cancer at baseline endoscopy. Participants received high-resolution endoscopy with targeted and systematic random biopsies following Cambridge protocol. Multivariable negative binomial regression, logistic regression, and linear mixed-effects models were applied to assess predictors of SRCC burden and temporal dynamics of SRCC detection.

**Findings:**

In PTG cohort, 89% individuals had early-stage cancer (pT1aN0M0) and 11% had no evidence of carcinoma (pT0N0M0). The number of SRCC foci ranged from 0 to 273 (median 14, IQR 2–37). The number of positive targeted and random biopsies independently predicted SRCC burden on gastrectomy (*P* < 0.001), whereas age, CDH1 mutation type and number of affected relatives were not significantly associated. In the surveillance cohort, the number of positive biopsies remained largely stable over time, with no significant temporal increase detected (Incidence rate ratio: 1.026 per six months, 95% CI: 0.985–1.068, *P* = 0.214).

**Interpretation:**

Endoscopic surveillance using systematic biopsies reliably estimates SRCC burden on gastrectomy in HDGC. Individuals with few SRCC lesions showed stable findings during long-term surveillance, supporting the safety of endoscopic monitoring and extended surveillance intervals. Future research should clarify the clinical significance of high SRCC burden.

**Funding:**

Medical Research Council and Cancer Research UK.

**Research in context**

**Evidence before this study**

We systematically reviewed published evidence on the management of hereditary diffuse gastric cancer (HDGC) in *CDH1* germline pathogenic variant (GPV) carriers. We searched PubMed and Google Scholar for articles published from January 1st 2000 to December 31st 2025 using the terms: “hereditary diffuse gastric cancer”, “endoscopic surveillance”, and “CDH1 mutation”. Relevant publications and international consensus and guidelines were examined.

Existing evidence shows that pathogenic *CDH1* GPV confers a high lifetime risk of diffuse gastric cancer. International guidelines recommend prophylactic total gastrectomy (PTG) as the primary risk-reduction strategy in patients with cancer findings on endoscopic biopsies. In recent years, emerging evidence show that endoscopic surveillance with systematic biopsies can help delay or even avoid surgery. Therefore, more recent US guidelines suggest a choice between PTG and endoscopic surveillance in cases with no evidence of advanced cancer. However, reliable predictors of early cancer burden are lacking and there is very limited evidence on the long term safety of endosopic surveillance in patients with endoscopic evidence of T1a lesions. In *CDH1 GPV* individuals, T1a lesions are common and often indolent, in contrast to early sporadic gastric cancers, which typically progress to advanced cancer. Data on optimal endoscopic surveillance intervals in this group of patients is missing.

**Added value of this study**

This study adds value by correlating endoscopic biopsy findings directly with pathological results from PTG specimens. We demonstrate that the number of endoscopic biopsies positive for SRCC independently predicts the total SRCC burden. We found that the combination of random and targeted biopsies provide the best prediction, offering clinicians a framework for shared decision making. Furthermore, our longitudinal analysis of nearly 500 gastroscopies across 20 years provides new evidence that endoscopic findings of SRCC foci remain stable over time in patients with few early cancer lesions. We provide compelling evidence on the indolent nature of a limited number of small T1a lesions and clinical safety of delaying surveillance intervals in this subgroup of patients.

**Implications of all the available evidence**

Current practice often favours early PTG for HDGC individuals with SRCC detected due to uncertainty regarding progression risk. Our findings suggest that systematic biopsies can accurately estimate cancer burden and identify individuals with low SRCC burden who remain stable during surveillance. In this subgroup, endoscopic surveillance with extended intervals is safe and could help delay or avoid surgery while maintaining oncological safety. Future research should clarify whether individuals with high SRCC burden are at heightened risk of diffuse gastric cancer.

## Introduction

Hereditary diffuse gastric cancer (HDGC) is an autosomal dominant cancer syndrome linked to germline pathogenic variants (GPV) of *CDH1* gene, that predisposes to diffuse gastric and lobular breast cancer.^1^ The lifetime risk of gastric cancer is estimated to be 7–42%; these estimates have significantly declined in the last decade as germline genetic testing including multigene panels have become more widespread in turn leading to a reduction in ascertainment bias.^2^^,^^3^ Most recent guidelines recommend offering the option of prophylactic total gastrectomy (PTG) or endoscopic surveillance for *CDH1*-GPV carriers who have no features of advanced cancer, but do not provide clear guidance in patients with early SRCC due to the paucity of data about the natural history of T1a lesions.^4^^,^^5^ In CDH1 GPV individuals, T1a lesions are common and often indolent, in contrast to early sporadic gastric cancers, which typically progress to advanced cancer.^2^, ^3^, ^4^, ^5^ Total gastrectomy is a life changing operation, which carries significant impact on eating behaviour and body image perception, with 80% of patients having at least one GI symptom at 12 months from the operation.^6^ Recent studies have challenged the recommendation to surgery in this group of people, indicating that, while signet ring cell carcinoma (SRCC) foci are seen in the vast majority of patients with HDGC, progression to advanced cancer is rare under active endoscopic surveillance.^7^, ^8^, ^9^ In these studies cancer stage 2 or higher occurred only in 2 out of 212 surveyed patients, who declined the offer of surgery on the basis of endoscopic findings.^7^^,^^8^ Lee et al., also provided early evidence that some individuals with positive endoscopic biopsies, even in the presence of subtle endoscopically visible lesions, can be safely monitored for several years, without clinical progression of the disease.^8^ This implies that T1a SRCC lesions in HDGC can be indolent, distinct from advanced disease, and do not all mirror the clinical features of early sporadic diffuse gastric cancer. However, analysis of PTG specimens shows a great range in the number of foci from none or very few to more than 200, suggesting that even in the context of pT1a disease, the individual risk of progression to advanced cancer could be very different.^10^ Therefore, uncertainty remains regarding suitability and timing of endoscopic monitoring in this group of individuals, as there are no reliable methods to risk stratify *CDH1-GPV* carriers and there are limited data to inform surveillance intervals. We have previously described and validated endoscopic criteria for detection and characterisation of early lesions, to provide endoscopists with a tool to improve quality of the endoscopic examination and confidence in the endoscopic diagnosis.^10^ In this study we aim to identify predictors of early SRCC burden in the stomach and provide evidence to tailor surveillance intervals based on clinical findings with particular focus on individuals with few SRCC lesions on endoscopic examination.

## Methods

### Cohorts, study design and ethics

In this study, two prospective longtitudinal cohorts of patients with HDGC and pathogenic or likely pathogenic germline *CDH1* (GPV) variants were included. Data were prospectively collected with full retrospective collection to integrate any missing data. Baseline information was recorded at enrolment. Gender information derived from digital medical records where patients self reported it. This study was approved by Cambridge South Research Ethics Committee (MREC97/5/32). Written informed consent was obtained from all participants involved in the study.

#### PTG cohort

**Fig. 1:**
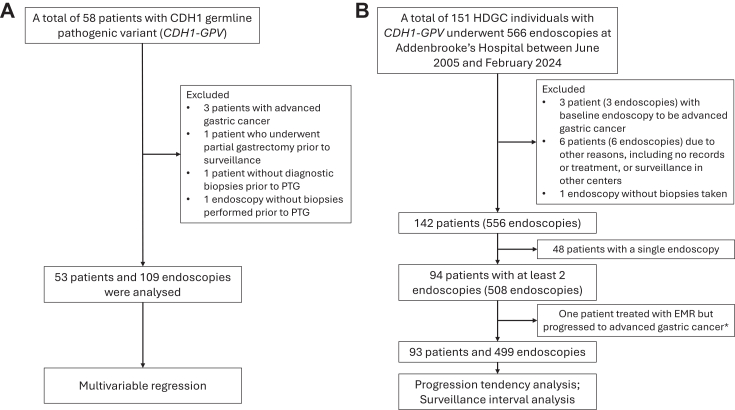
**Study cohort flowcharts.** (A) Flowchart outlining inclusion and exclusion criteria for the PTG (prophylactic total gastrectomy) cohort. (B) Flowchart for the endoscopy cohort. From 151 individuals who underwent 566 surveillance endoscopies, exclusions were made for negative advanced cancer at baseline, incomplete records, and single-timepoint cases, resulting in 93 individuals and 499 endoscopies. Notably, one patient was excluded due to endoscopic intervention that deviated from the surveillance pathway (see Discussion for extensive description of this case).

This consisted of 53 *CDH1*-GPV individuals who underwent at least one endoscopy followed by PTG at Addenbrooke's Hospital between August 2005 and August 2024. We included endoscopic procedures with mapping biopsies according to the Cambridge protocol in the analysis.^4^^,^^8^^,^^11^ We excluded individuals or endoscopies without diagnostic biopsies prior to PTG, and patients whose management deviated from guideline recommendations (endoscopic resection or partial gastrectomy). Data were analysed from first surveillance endoscopy to the date of the gastrectomy. The total number of SRCC foci, defined as invasive carcinoma at least into the lamina propria (excluding pagetoid lesions), was meticulously assessed to measure cancer burden across the entire gastrectomy specimen by gastrointestinal pathologists with special expertise in HDGC. The gastrectomy specimen was opened along the greater curvature in the fresh state, pinned on to cork board and formalin fixed for 24 h. After fixation, resections margins were embedded and the specimen was split into anterior and posterior segments. Each segment was then divided into anatomical regions (cardia, fundus, transition zone, antrum and pylorus). The entire specimen was paraffin embedded with alternate sections kept for research. All accompanying lymph nodes were also embedded. This cohort was primarily used to explore the relationship between endoscopic findings and PTG (Fig. 1A).

#### Endoscopy surveillance cohort

This consisted of 93 *CDH1*-GPV individuals who underwent a combined total of 499 endoscopic surveillance procedures at Addenbrooke's Hospital between June 2005 and February 2024. The exclusion criteria were as follows: baseline endoscopy revealing macroscopic evidence of invasive cancer as these patients proceeded directly to treatment rather than surveillance, lack of clinical records or incomplete surveillance data, use of endoscopic resection which altered the endoscopy SRCC burden and individuals with single surveillance endoscopy (Fig. 1B). This cohort is analysed for long-term disease progression of HDGC individuals under active surveillance until the last surveillance endoscopy. Patients in PTG cohort who were under surveillance are included in the endoscopy cohort. Patient characteristics of the two cohorts are summarised in Table 1.

**Table 1:** The baseline characteristics of patients in the cohorts of prophylactic total gastrectomy and endoscopic surveillance.

Characteristics	PTG cohort (n = 53)	Endoscopic surveillance cohort (n = 93)
Age at PTG or study cutoff, years, median (IQR)	31.0 (24–41)	40.6 (31.5–54.6)
Gender, Numbers (percentage)		
Female	26 (49.1%)	48 (51.6%)
Male	27 (50.9%)	45 (48.4%)
Ethnicity, Numbers (percentage)		
White	43 (81.1%)	76 (81.7%)
Asian	5 (9.4%)	11 (11.8%)
Others	5 (9.4%)	6 (6.5%)
Number of endoscopies, median (IQR, range)	3 (1–5, 1–8)	4 (3–6, 2–15)
Follow-up time (months), median (IQR, range)	33 (15–47, 1–88)	39 (20–64, 4–159)
CDH1 mutation, Numbers (percentage)^a^		
Truncating	27 (50.9%)	–
Frameshift	11 (20.8%)	–
Splice	6 (11.3%)	–
Missense	5 (9.4%)	–
Unknown	4 (7.5%)	–
Number of 1st or 2nd degree relatives with symptomatic gastric cancer		
0	16 (30.2%)	–
1	15 (28.3%)	–
2	17 (32.1%)	–
3	4 (7.5%)	–
4	1 (1.9%)	–
Stage, Numbers (percentage)		
pT1aN0M0	47 (89%)	–
pT0N0M0	6 (11%)	–
Number of intramucosal SRCC foci, median (range)	13 (0, 273)	–

a CDH1 mutation subtypes were not analysed in the endoscopic surveillance cohort because the variants were recorded only as pathogenic, likely pathogenic, variants of uncertain significance, or benign in the original data collection.

### Procedures

Endoscopies were done with a high-resolution endoscope with optical magnification (Lucera CV-260, Lucera CV-290 and EVIS ×1, Olympus, Tokyo, Japan). White-light imaging and narrow band imaging were used to inspect all anatomical regions of a well insufflated stomach. During endoscopies, targeted biopsies were taken from visible lesions, including pale areas, erosion, erythematous areas, ulcers or polyps. Random biopsies were performed according to the Cambridge protocol (four to five biopsy specimens in cardia, fundus, corpus, transitional zone, antrum, and pre-pyloric area, respectively) before May 1, 2019. After this date, a modified Cambridge protocol was adopted, taking two biopsies in the pre-pyloric area, four biopsies in the antrum, transitional zone, fundus, and cardia; and six biopsies in the corpus.^4^^,^^5^^,^^8^^,^^11^ Targeted biopsies were obtained as single bites per pass. Random biopsies were taken as double bites per pass after the randomised controlled trial reported in 2021.^12^ Biopsy specimens were stained with haematoxylin and eosin (and periodic acid-Schiff diastase at discretion of the reporting pathologist) to assess for the presence of SRCC foci. Two senior endoscopists and three endoscopy fellows trained in advanced imaging performed the endoscopic procedures. All cases were reviewed by upper gastrointestinal specialist pathologists with extensive experience in the identification of SRCC.

### Multivariable regression analysis and sensitivity analysis

**Fig. 2:**
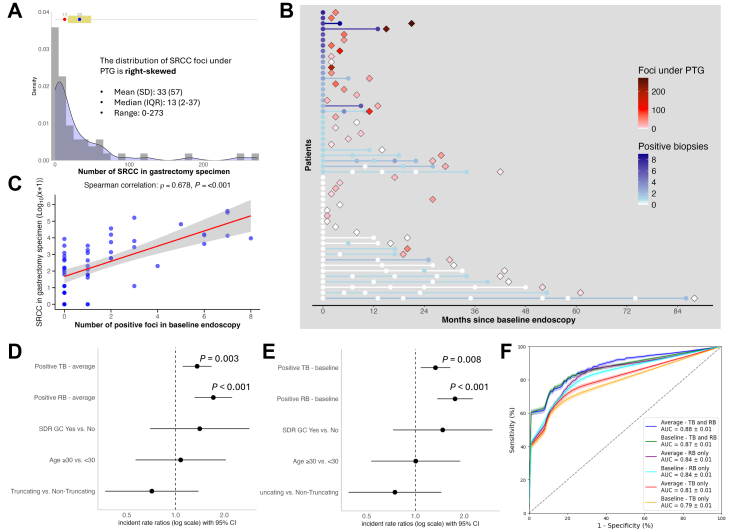
**The association of endoscopic findings and cancer burden at prophylactic total gastrectomy (PTG). A. Distribution of Signet ring cell carcinoma (SRCC) foci in PTG specimens.** Histogram showing the right-skewed distribution of SRCC foci counts among PTG patients. The majority had fewer than 50 foci, but a subset exhibited extremely high burden (>200 foci). **B. Association between endoscopic biopsy findings and signet ring cell carcinoma (SRCC) burden in gastrectomy specimens.** Each row represents one individual who underwent endoscopic surveillance followed by PTG, and the lines were coloured by the number of positive biopsies in the last endoscopy. Circles denote endoscopic procedures, coloured by the number of positive biopsies, while diamonds represent PTG events, coloured by the total number of SRCC foci identified in the gastrectomy specimens. The most recent endoscopies were excluded for two patients (the second and second-to-last cases in the figure), as no biopsies were taken during their final procedure prior to PTG. A few patients without positive endoscopic biopsies underwent PTG based on their personal willingness. **C. Correlation between the number of positive foci detected at baseline endoscopy and the number of early SRCC foci identified in PTG specimens.** Spearman correlation analysis demonstrated a moderate association, with coefficient value of 0.678 (*P* < 0.001). **D. Multivariable regression model identifying independent predictors of SRCC burden.** Negative binomial regression using the average number of positive targeted biopsies (TB) and random biopsies (RB) per patient showed both TB (*P* = 0.003) and RB (*P* < 0.001) were significant predictors of SRCC foci in PTG. **E. Regression analysis restricted to findings from the baseline endoscopy only.** Both TB (*P* = 0.008) and RB (*P* < 0.001) remained independent predictors. SDR: First and Secondary degree of relatives. **F. Logistic regression models trained to classify patients into high (≥30 foci) or low (≤20 foci) burden categories.** The model showed the best performance (AUC = 0.88) when both TB and RB were included. AUC were presented as the average ± 95% CI across 1000 bootstrap iterations.

To identify independent predictors of SRCC burden in prophylactic total gastrectomy (PTG) specimens, we applied a multivariable negative binomial regression model, since the distribution of SRCC foci is skewed (Fig. 2A). The response variable was the total number of signet ring cell carcinoma (SRCC) foci identified by histopathological assessment of PTG specimens. Prespecified variables included patient age group at baseline, CDH1 mutation type (truncating vs non-truncating), if 1st and 2nd degree relatives (SDRs) were died from symptomatic clinical gastric cancer (identified from pedigree analysis documented in the clinical genetics records), and endoscopic findings. For endoscopic predictors, we calculated the mean number of positive targeted biopsies (TB) and random biopsies (RB) across all endoscopies prior to PTG. A second model was also fitted using only baseline endoscopy findings.

To evaluate the robustness of our primary findings, we performed several post-hoc sensitivity analyses within the negative binomial regression framework. In the main model, the number of positive TB and RB were included as continuous count variables, family history (number of first-degree relatives who died from gastric cancer) was included as a binary variable, and age and mutation type were modelled as categorical variables. To assess the stability of the model, we explored alternative functional forms of key predictors. First, age was modelled as a continuous variable (per year) rather than a dichotomous variable (<30 vs ≥ 30 years) to avoid potential information loss due to arbitrary categorisation. Second, family history was modelled as a continuous count variable to assess a potential dose–response relationship. Third, biopsy findings were modelled as binary indicators (positive vs negative) instead of continuous counts to evaluate whether the presence of positive biopsies, rather than the number of positive samples, influenced the results.

Furthermore, to assess whether the observed associations were driven by influential observations, we performed an outlier diagnostic analysis. Cook's distance was calculated for each observation in the primary model to evaluate influence on regression estimates. The negative binomial model was then refitted after excluding the most influential observations (those with the highest Cook's distance values).

Results from all sensitivity analyses were reported as incidence rate ratios (IRRs) with 95% Wald confidence intervals (CIs) and corresponding p-values. Consistency in effect direction and statistical significance across models was considered evidence of robustness.

To evaluate the predictive utility of the endoscopic variables, logistic regression models were trained to classify patients into low (≤20 foci, n = 32) or high (>30 foci, n = 16) SRCC burden groups. These prespecified thresholds were selected based on the observed right-skewed distribution of SRCC foci in gastrectomy specimens and to achieve an approximate 2:1 ratio between low- and high-burden groups. Patients with an intermediate burden (20–30 foci, n = 5) were excluded to avoid arbitrary classification within this grey zone defined by the data distribution. Models were developed using factors consistenly and significantly independent associated with SRCC burden. Performance was assessed using area under the receiver operating characteristic curve (AUC, presented as average ± 95% CI) over 1000 bootstrap iterations.

### Temporal trends for biopsy findings in endoscopic surveillance cohort

To investigate longitudinal changes in biopsy-detected SRCC foci, we applied mixed-effects regression models using negative binomial distribution to accommodate overdispersion in count data. The prespecified primary outcome was the number of positive biopsies (random and targeted) per endoscopy. The main predictor was months since baseline endoscopy, with a random intercept for each patient to account for within-subject correlation across repeated surveillance.

### Surveillance interval analysis

In our cohort, surveillance endoscopy was performed at 6 month intervals if SRCC was detected, 12 months interval for patients with negative findings. To evaluate the safety and effectiveness of different surveillance intervals for individuals with low number of positive biopsies, we looked at the outcomes of patients who had 1–2 positive biopsies at any endoscopy and subsequently underwent at least one further surveillance procedure. To simulate annual or biennal surveillance intervals, we masked examinations performed within the defined interval. This allowed to observe what SRCC findings would look like if patients were reviewed annually or every two years. For each interval, biopsy findings were compared to those from the immediately preceding endoscopy. Progression status was defined as follows: Progressed: an increase by more than one positive biopsy; Stable: no change or an increase by only one positive biopsy; and Regressed: a decrease in the number of positive biopsies. The proportions of patients in each category were calculated and visualised to evaluate temporal patterns and the effectiveness of different surveillance intervals.

### Statistical analysis

All statistical analyses were performed using R (version 4.5.1). Descriptive statistics for continuous variables are presented as medians with interquartile ranges (IQR), while categorical variables are summarised as frequencies and percentages. Group comparisons for continuous data were conducted using the Wilcoxon rank-sum test. Multivariable negative binomial regression models were fitted using the MASS package, while mixed-effects negative binomial regressions incorporating random intercepts were performed using the glmmTMB package. Model extraction, tidying, and evaluation were facilitated by the broom, broom. mixed, and purrr packages. Logistic regression performance were evaluated with AUC over 1000 bootstrap resamples to derive robust internal validation metrics and 95%CI. A two-sided p-value of <0.05 was considered statistically significant for all analyses. There was no pre-specified sample size.

### Role of the funding source

The funding source had no role in the study design, collection, analysis, or interpretation of the data and the writing of the report.

## Results

### Endoscopic surveillance findings and cancer burden gastrectomy specimen (PTG cohort)

The flowchart and patient characteristics of the 53 individuals who received PTG are shown in Fig. 1A, Figure S1 and Table 1. The cohort had a median age of 31.0 years at the time of PTG (IQR: 24–41, range: 19–63) and was evenly distributed by gender (49% female, 51% male). Most patients were of White ethnicity (81%), and truncating germline PV accounted for 51% of all *CDH1-PV*. The number of intramucosal SRCC foci identified in PTG specimens ranged from 0 to 273, with a median of 13 foci (Table 1).

To explore the relationship between surveillance findings and cancer burden, we visualised each patient's endoscopic results alongside the number of SRCC foci identified in their PTG specimens (Fig. 2B). Patients were ordered by the number of positive biopsies detected during baseline endoscopy. This visualisation revealed that individuals with higher SRCC burden were predominantly those with positive findings at baseline endoscopy identified by random and targeted biopsies. When considering only random or only targeted biopsies, the total burden could be under-estimated with more high-burden cases with a low number of SRCC lesions at endoscopy (Figure S2). Histogram showing the right-skewed distribution of SRCC foci counts from PTG specimens. The majority (81% 43/53) had no more than 50 foci, but a subset exhibited extremely high burden [4% (2/53) >200 foci] (Fig. 2A).

To quantify this observation, we assessed the correlation between the number of positive foci detected at baseline endoscopy and the number of early SRCC foci identified in PTG. Spearman correlation analysis demonstrated a moderate association, with coefficient value to be 0.678 (*P* < 0.001) (Fig. 2C).

### Clinical predictors of SRCC burden in the PTG cohort

To evaluate the predictive value of endoscopic, clinical, and genomic factors for SRCC burden, we included age, the number of SDR who died from gastric cancer, and CDH1 mutation type (truncating vs non-truncating) in subsequent analyses. In multivariable negative binomial regression using averaged endoscopy data (Fig. 2D), both the number of positive targeted biopsies (*P* = 0.003) and random biopsies (*P* < 0.001) were significantly associated with higher SRCC burden. When restricting the model to baseline endoscopy data (Fig. 2E), these two predictors remained significant (*P* = 0.008 and *P* < 0.001, respectively). Family history (number of SDR who died from gastric cancer) showed a borderline significant association in the sensitivity analyses but was insignificant in the main model (Tables S1 and S2). None of the other clinical variables, including age and CDH1 mutation type, were independently associated with SRCC burden in either model. The SRCC burden stratified by clinical and endoscopic predictors are shown in Table S3.

Logistic regression models incorporating both mean TB and RB positivity achieved the best classification performance for identifying patients with high SRCC burden (Fig. 2F). This model yielded an AUC of 0.88 ± 0.01. A model using only baseline TB and RB also performed well (AUC = 0.87 ± 0.01). In contrast, models using TB or RB alone showed lower performance. TB-only models had reduced sensitivity (AUC = 0.81 ± 0.01 for average positivity, 0.79 ± 0.01 for baseline positivity), while RB-only models achieved intermediate results (AUC = 0.84 ± 0.01 for both average and baseline positivity), underscoring the importance of combining both biopsy strategies in surveillance.

### Temporal trends of SRCC detection in the endoscopy surveillance cohort

**Fig. 3:**
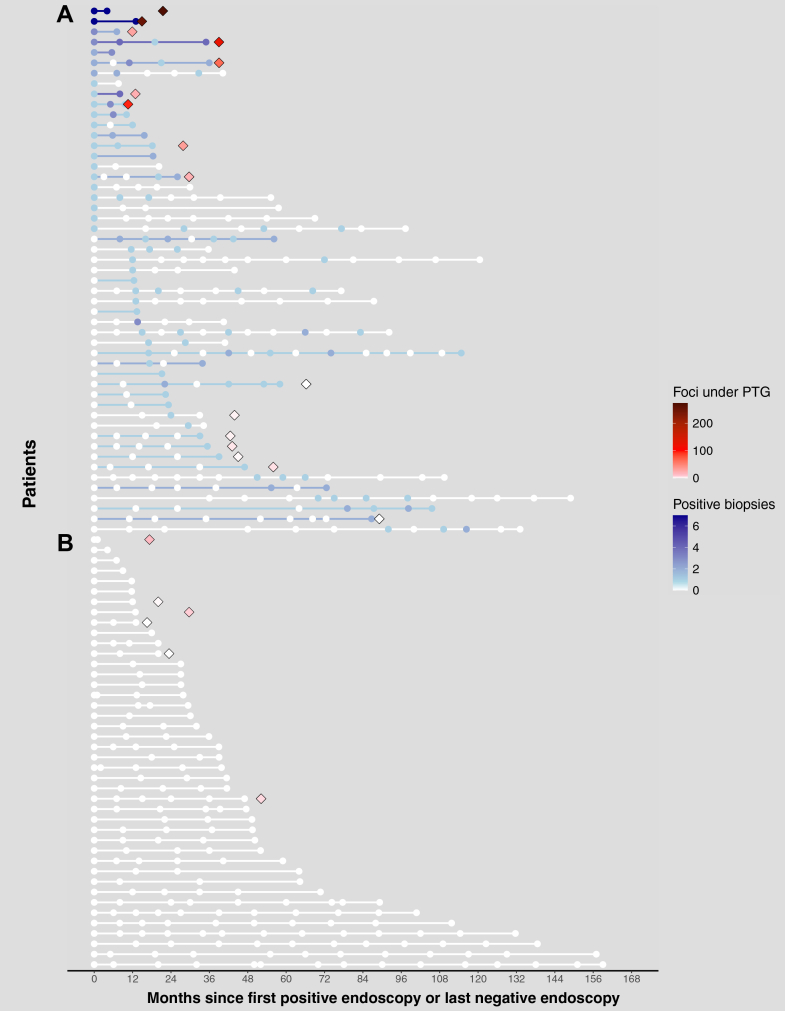
**Longitudinal endoscopic findings in CDH1 germline pathogenic variant (GPV) carriers undergoing surveillance endoscopy.** (A) Individuals with positive biopsies during endoscopy surveillance (n = 51). Overall median (mean) follow-up: 40.2 (48.0) months over 5 (5.6) endoscopies; after the first positive detection: 20.2 (30.2) months over 3 (4.1) endoscopies; before the first positive detection: 12.0 (17.8) months over 1 (1.5) endoscopies. (B) Individuals without positive biopsies during endoscopy surveillance (n = 42). Overall median (mean) follow-up: 39.0 (49.0) months over 4 (5.0) endoscopies. Each row represents one individual who underwent endoscopic surveillance. Circles denote endoscopic procedures, coloured by the number of positive biopsies (both random and targeted), with separated counts shown in Figure S4; diamonds represent prophylactic total gastrectomy (PTG) events, coloured by the total number of SRCC foci identified; the lines were coloured by the number of positive biopsies in the last endoscopy. The most recent endoscopy were excluded for one patients (the top case in the figure), as no biopsies were taken during their final procedure prior to PTG. A few patients without positive endoscopic biopsies underwent PTG based on their personal willingness. Endoscopy follow-up ended either at the time of PTG or by February 2024. Endoscopic findings remained largely stable over time, showing fluctuations rather than consistent progression.

A total of 499 endoscopic procedures from 93 *CDH1-GPV* carriers from the endoscopy cohort were analysed to evaluate temporal trends in SRCC detection. Patient inclusion and exclusion flowchart was shown in Fig. 1B. The median follow-up time was 39 months (IQR: 20–64, maximum 159), with a median of 4 endoscopies per patient (IQR: 3–7, maximum 15). Among these individuals, 42 (45%) had no SRCC detected throughout surveillance [median follow-up 39 months (IQR: 21–57, maximum 159); median 4 endoscopies (IQR: 3–6, maximum 15)]; 46 patients (49%) had at most 1–2 positive biopsies (all lesions size <10 mm) through a median follow-up of 41 months (IQR: 21–72, maximum 148) and a median of 4 endoscopies (IQR: 3–6, maximum 15)]; 5 patients has ≥3 positive biopsies and received a shorter median follow-up of 15 months (IQR: 11–39, maximum 40) and a median 2 endoscopies (IQR: 2–4, maximum 6)] (Figure S3). The median age at first detection of SRCC during endoscopy surveillance was 32.5 y (25–47 y, range 19–80). The endoscopic findings appeared largely stable over time, with fluctuations rather than consistent progression (Fig. 3). When random and targeted biopsies were presented separately, similar patterns were observed (Figure S4). Quantitative analysis with mixed-effects negative binomial regression confirmed the absence of a significant longitudinal trend in SRCC detection. The number of positive biopsies in surveillance endoscopy did not significantly increase over time (IRR: 1.026 per six months, 95% CI: 0.985–1.068, *P* = 0.214) across the entire surveillance cohort. Further analysis stratified by baseline endoscopic findings showed non-significant trend toward an increase in the number of positive biopsies over time (IRR: 1.058 per six months, 95% CI: 0.998–1.122, 95% CI: 0.784–0.990, *P* = 0.058) in patients with negative baseline endoscopy (Figure S5). Conversely, patients who had positive biopsies at baseline demonstrated a significant decrease in the number of positive biopsies during follow-up (IRR: 0.881 per six months, 95% CI: 0.784–0.990, *P* = 0.004). This likely reflects the inherent sampling challenges of endoscopic surveillance, rather than a true biological regression of the disease. These findings suggest that disease progression in HDGC is generally slow, with endoscopic findings characterised by temporal variability rather than steady progression.

### Endoscopic findings across various surveillance intervals

Among the surveillance cohort, 46 individuals had 1–2 positive biopsies at their first positive examination, among whom 36 subsequently underwent at least one further surveillance procedure. In our cohort, surveillance endoscopy was at 6 month intervals if SRC was detected. To evaluate the effect of different surveillance intervals (6, 12, or 24 months), we simulated longer surveillance schedules by masking examinations performed within the defined interval.

The majority of subsequent surveillance procedures demonstrated stable findings: 62.2% (92/148) at 6-month intervals, 65.9% (81/123) at 12 months, and 67.6% (50/74) at 24 months. A third of these patients (31%–32%) exhibited decrease in the number of SRCC positive biopsies, highlighting some degree of variability in biopsy outcomes over time. There was no progression to advanced cancer in any of those patients (pT1a or earlier), and are currently alive without disease-related mortality. An increase in endoscopically detected SRCC foci was infrequent, occurring in only <6% of cases across all intervals (Figure S6). Nevertheless, 19.4% (7/36) of these patients received PTG either based on physician advice due to increased number of positive biopsies on endoscopic biopsies (n = 2) or due to personal preference (n = 5).

## Discussion

This study demonstrates that in patients with HDGC it is possible to estimate the burden of early SRCC based on the integration of histopathology data from random and targeted biopsies. In addition, in a well-characterised longitudinal cohort of patients with no endoscopic features of advanced cancer and low endoscopy SRCC burden, we demonstrate that endoscopic surveillance is safe and increase in the number of early cancer lesions is an infrequent event.

Given the aggressive nature of diffuse gastric cancer and the evidence that early SRCC can be endoscopically inconspicuous, PTG has been for decades the mainstay of treatment. This has led to the recommendation of surgery upon detection of SRCC on endoscopic biopsies.^13^, ^14^, ^15^ However, findings challenge a blanket policy of major surgery for individuals with limited number of SRCC foci, as these lesions can be indolent. Several case series and two larger longitudinal cohort studies have in fact demonstrated that progression to advanced cancer on surveillance is a rare event.^7^^,^^8^^,^^16^ Given the rarity of progression to Stage >1 cancer, it is difficult to develop prediction models for progression to advanced cancer. Although imperfect, this strategy is conceptually similar to risk-stratification strategies used in other gastrointestinal conditions characterised with marked spatial heterogeneity, such as Barrett's oesophagus, gastric atrophy and ulcerative colitis. In these diseases there is evidence that multiple independent clones can co-exist within the pre-invasive lesions while the topographical extent of the disease correlates with the risk of cancer.^17^, ^18^, ^19^, ^20^, ^21^ Our data show that the number of SRCC foci on both random and targeted biopsies are strong predictors of early cancer burden and that the combination of the two types of biopsies achieves the best diagnostic accuracy. Although family history was demonstrated to be significantly associated with the cumulative risk of gastric cancer in HDGC families,^2^^,^^3^ our data showed borderline significant association in sensitivity analyses but not in the main model, likely reflecting the small sample size and the prospective surveillance design, in which individuals with a stronger family history may be more likely to elect PTG regardless of disease burden. This study specifically evaluated outcomes in patients with positive endoscopic biopsies. In most previously published case series, this topic was either not addressed directly or difficult to resolve as patients with positive findings proceeded to PTG or had short follow up time.^7^^,^^8^^,^^14^, ^15^, ^16^ Our findings demonstrate that a low endoscopic disease burden correlates with both a low burden of early cancer in gastrectomy specimens and a low risk of progression during surveillance, therefore providing important evidence to inform clinical management.

It has been debated whether mapping biopsies are useful in the monitoring of these individuals, with the argument that it is clinically insignificant to detect indolent microscopic foci of disease, given that around 90% of *CDH1-PV* carriers have microscopic foci detected in PTG specimens.^22^ However, random sampling appears clinically important because it provides a standardised, endoscopist-independent method of diagnosing microscopic disease that is often endoscopically occult, particularly in the context of general endoscopy practice. Accurate endoscopic identification of SRCC or other high-risk lesions is inherently challenging given their subtle or absent mucosal changes, as reflected by the large variation in the rate of positive targeted biopsies reported in the literature (Table S4).^23^ We acknowledge that repeated random sampling increase procedure time and pathology workload, and could lead to mucosal scarring that might obscure subtle lesions. Therefore, the advantages and disadvantages of random sampling should be weighed. The previously published Cambridge criteria help distinguish benign scar from SRCC-associated abnormalities, which can mitigate the risk of that scar can hinder assessment of gastric mucosa.^10^

One case, who was excluded from the analysis due to deviation from guidelines recommendation, helps appreciate the role of random biopsies. This patient was recommended therapeutic gastrectomy due detection of a high risk 15 mm flat lesion with confirmed SRCC on targeted biopsy. As the patient refused therapeutic gastrectomy, a diagnostic endoscopic resection was performed, which showed R0 resection of a T1a signet ring cells carcinoma with no lymph-vascular invasion. Three subsequent surveillance endoscopies at 6-month intervals showed no visible mucosal lesions and no positive RB. However, at the fourth surveillance endoscopy three SRCC foci were found on RB in the absence of a macroscopically visible lesion. The patient subsequently underwent gastrectomy, which revealed extensive tumour involvement in the submucosa, muscularis propria, and subserosa (99 of 110 tissue blocks positive), with minimal mucosal involvement (pT4aN1M0). The patient then received adjuvant chemotherapy and died eight years after treatment. This case illustrates the utility of random sampling in the presence of normal appearing mucosa due to the biological possibility of deep-layer tumour involvement with minimal surface disease.

**Fig. 4:**
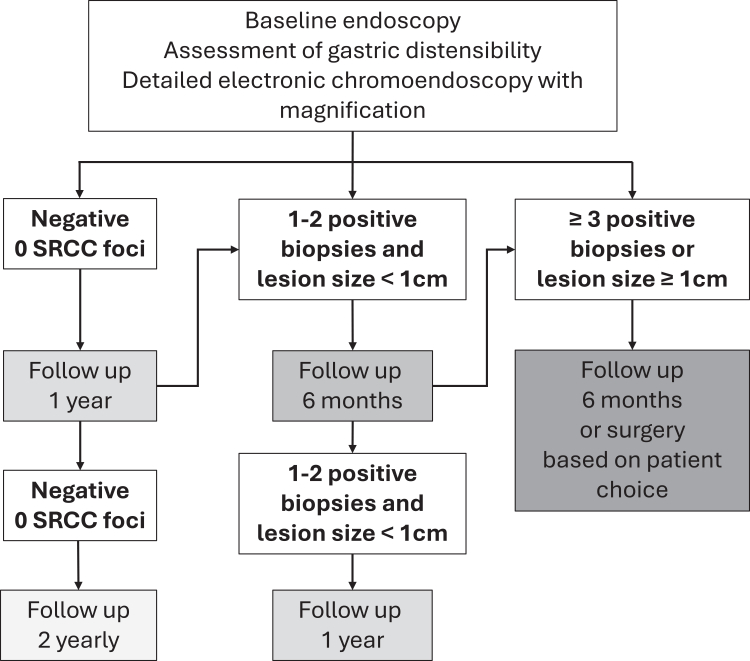
**A modified surveillance protocol for *CDH1* germline pathogenic variant carriers, supported by evidence of safety from the study.** The modified surveillance intervals should only be followed if taking both targeted and random mapping biopsies with Cambridge protocol.

Current guidelines recommend that individuals with positive biopsy findings, who decline PTG, be seen for repeat OGD in 6 months.^4^ Our data not only demonstrate the safety of endoscopic monitoring even in the presence of targeted or random biopsies positive for SRCC, within the context of informed patient choice and multidisciplinary review, but also suggest that current intervals for surveillance are too short in patients with 1–2 positive biopsies and early follow up unlikely to provide clinically useful information. In particular, our data indicate that a negative baseline endoscopy based on full mapping biopsies and high quality endoscopy with electronic chromoendoscopy and magnification is a strong predictor of low burden of disease meaning that follow up could be extended to in expert centres. Also, the findings suggest that a six month follow up is not necessary in the presence of a small number of biopsies positive for SRCC and in the absence of worrying endoscopic features (elevated or depressed lesions, ulcers or thickened folds).^17^ Based on the data, annual surveillance with Cambridge protocol mapping biopsies appears safe and reasonable for patients with low endoscopic cancer burden, who demonstrate largely stable endoscopy findings during surveillance. Consideration to extend interval to 2 years after multiple consecutive negative endoscopies could be given in selected cases, such as those with no visible lesions (Fig. 4). In contrast, the natural history of higher-burden disease (≥3 positive biopsies during surveillance endoscopy) remains elusive because it was curtailed in this study by gastrectomy. Notably, while the distinction between 1 and 2 vs ≥3 positive biopsies might seems arbitrary, it is supported by the observation that patients with ≥3 positive biopsies on endoscopy were typically associated with substantially higher SRCC burden in gastrectomy specimens. Future multi-centre studies in patients with high number of endoscopic SRCC lesions declining surgery might provide an opportunity to further adjust of surveillance intervals.

This study has several limitations. First, although we present long follow up data from a longitudinal cohort of CDH1 GPV carriers with positive endoscopic findings, the overall sample size remains modest, particularly in the PTG cohort used to correlate endoscopic findings with gastrectomy pathology. Second, investigating the outcomes of patients with higher number of positive endoscopic biospies was not possible because they typically proceeded to gastrectomy shortly after detection, limiting the opportunity to observe the natural history of higher-burden disease under surveillance. The observed stability in the number of SRCC lesion over time should not be interpreted as direct evidence for a simple continuum low burden to high burden to advanced cancer. Future studies should address the risk of advanced cancer risk in patient with high early SRCC burden. Third, the cohort was derived from a single tertiary referral centre and consisted predominantly of individuals of European ancestry, which reflects the demographics of most published HDGC surveillance cohorts but may limit generalisability to other populations.

Taken together our findings suggest that SRCC progression is generally uncommon under surveillance, and low endoscopic SRCC burden correlates with scarce early neoplastic gastric disease. This study provides a framework for shared decision making in patients with endoscopic evidence of early SRCC.

## Contributors

LW collected and analysed the data and wrote the manuscript. HJL collected the data and contributed to analysis and design. NK and SS assisted data collection. MO’D directed histopathological assessment of hereditary diffuse gastric cancer samples. MT provided input on genetic data. RON performed PTG and provided data. RCF is chief investigator on the Familial Gastric Cancer Registry study, conceived the biopsy protocol, provided guidance on the project. MdP conceived the project, performed endoscopic procedures, conceived the endoscopic diagnostic criteria, and wrote the manuscript. LW and MdP accessed and verified the underlying data. The corresponding author had final responsibility for the decision to submit the manuscript for publication. All authors read and approved the manuscript before submission.

## Data sharing statement

Deidentified participant data with data dictionary will be shared upon reasonable request to the corresponding author.

## Declaration of interests

RCF is named on patents for Cytosponge™ and related biomarker assays including those licenced by the Medical Research Council to Medtronic (formerly Covidien) and Own shares in Cyted Health Ltd (Company No. 11478299). All other authors declare no competing interests.
