# Supplementary material for: Predictors of early cancer burden in CDH1 pathogenic variant carriers: a UK single-centre cohort study
Source: eClinicalMedicine. 2026 Jul 2;97:104036. doi: 10.1016/j.eclinm.2026.104036 (PMC13351748; doi:10.1016/j.eclinm.2026.104036)
Supplement: Supplementary Figures Tables [file mmc1.pdf]

Content list

Page 2: Supplementary Table S1

Page 3: Supplementary Table S2

Page 4: Supplementary Table S3

Page 5: Supplementary Table S4

Page 6: Supplementary Figure S1

Page 7: Supplementary Figure S2

Page 8: Supplementary Figure S3

Page 9: Supplementary Figure S4

Page 10: Supplementary Figure S5

Page 11: Supplementary Figure S6

Table S1.

Variable	Model 1 (Base), IRR (95% CI), p	Model 2 (Continuous Age), IRR (95% CI), p	Model 3 (Continuous GC Died), IRR (95% CI), p	Model 4 (Binary TB/RB), IRR (95% CI), p	Model 5 (Without the Top Outlier), IRR (95% CI), p
**Age - Category**					
<30	1 (Reference)	-	1 (Reference)	1 (Reference)	1 (Reference)
>=30	1.08 (0.57-2.04) (p=0.825)	-	1.08 (0.56-2.08) (p=0.815)	0.94 (0.47-1.90) (p=0.868)	0.86 (0.46-1.60) (p=0.626)
**Age - Continuous**	-	1.00 (0.97-1.03) (p=0.832)	-	-	-
**Mutation type**					
Non-Truncating	1 (Reference)	1 (Reference)	1 (Reference)	1 (Reference)	1 (Reference)
Truncating	0.71 (0.37-1.39) (p=0.319)	0.71 (0.36-1.37) (p=0.307)	0.73 (0.37-1.43) (p=0.353)	0.53 (0.26-1.05) (p=0.070)	0.61 (0.32-1.16) (p=0.131)
**GC deaths in SDR - Category**					
No	1 (Reference)	1 (Reference)	-	1 (Reference)	1 (Reference)
Yes	1.41 (0.70-2.86) (p=0.340)	1.38 (0.66-2.86) (p=0.390)	-	0.93 (0.44-1.95) (p=0.839)	2.01 (1.00-4.04) (p=0.049)
**GC deaths in SDR - Continuous**	-	-	1.09 (0.78-1.52) (p=0.624)	-	-
**TB average - Category**					
Negative	-	-	-	1 (Reference)	-
Positive	-	-	-	3.73 (1.87-7.44) (p=<0.001)	-
**TB average - Continuous**	1.36 (1.11-1.67) (p=0.004)	1.36 (1.10-1.67) (p=0.004)	1.38 (1.12-1.70) (p=0.003)	-	1.39 (1.14-1.69) (p=0.001)
**RB average - Category**					
Negative	-	-	-	1 (Reference)	-
Positive	-	-	-	2.69 (1.27-5.72) (p=0.010)	-
**RB average - Continuous**	1.71 (1.31-2.24) (p=<0.001)	1.72 (1.31-2.25) (p=<0.001)	1.75 (1.34-2.28) (p=<0.001)	-	1.77 (1.37-2.28) (p=<0.001)

SRCC, Signet ring cell carcinoma; PTG, Prophylactic total gastrectomy; TB: targeted biopsy; RB: Random biopsy.

Table S2.

Variable	Model 1 (Base), IRR (95% CI), p	Model 2 (Continuous Age), IRR (95% CI), p	Model 3 (Continuous GC Died), IRR (95% CI), p	Model 4 (Binary TB/RB), IRR (95% CI), p	Model 5 (Without the Top Outlier), IRR (95% CI), p
**Age - Category**					
<30	1 (Reference)	-	1 (Reference)	1 (Reference)	1 (Reference)
>=30	1.01 (0.53-1.89) (p=0.987)	-	0.99 (0.52-1.89) (p=0.978)	1.07 (0.57-2.03) (p=0.827)	0.80 (0.43-1.47) (p=0.469)
**Age - Continuous**	-	1.00 (0.97-1.03) (p=0.987)	-	-	-
**Mutation type**					
Non-Truncating	1 (Reference)	1 (Reference)	1 (Reference)	1 (Reference)	1 (Reference)
Truncating	0.75 (0.39-1.45) (p=0.387)	0.75 (0.39-1.45) (p=0.389)	0.76 (0.38-1.48) (p=0.415)	0.79 (0.41-1.55) (p=0.497)	0.64 (0.34-1.20) (p=0.160)
**GC deaths in SDR - Category**					
No	1 (Reference)	1 (Reference)	-	1 (Reference)	1 (Reference)
Yes	1.47 (0.73-2.99) (p=0.281)	1.48 (0.71-3.06) (p=0.295)	-	1.09 (0.54-2.20) (p=0.811)	2.12 (1.06-4.24) (p=0.034)
**GC deaths in SDR - Continuous**	-	-	1.09 (0.78-1.52) (p=0.631)	-	-
**TB baseline - Category**					
Negative	-	-	-	1 (Reference)	-
Positive	-	-	-	3.12 (1.58-6.17) (p=0.001)	-
**TB baseline - Continuous**	1.33 (1.08-1.64) (p=0.008)	1.33 (1.08-1.64) (p=0.008)	1.33 (1.08-1.65) (p=0.009)	-	1.35 (1.11-1.65) (p=0.003)
**RB baseline - Category**					
Negative	-	-	-	1 (Reference)	-
Positive	-	-	-	4.71 (2.40-9.22) (p=<0.001)	-
**RB baseline - Continuous**	1.76 (1.36-2.26) (p=<0.001)	1.76 (1.36-2.27) (p=<0.001)	1.81 (1.41-2.34) (p=<0.001)	-	1.82 (1.43-2.31) (p=<0.001)

SRCC, Signet ring cell carcinoma; PTG, Prophylactic total gastrectomy; TB: targeted biopsy; RB: Random biopsy.

Table S3.

Variable	N	Number of SRCC under PTG, Median (IQR)	Wilcoxon test P-value
Age at baseline			
<30 (Ref)	27	21.0 (3.5-47.0)	
>30	26	8.5 (2.0-20.0)	0.318
Mutation type			
Non_Truncating (Ref)	22	15.0 (5.5-59.0)	
Truncating	27	7.0 (1.5-29.0)	0.114
Unknown	4	29.0 (16.2-40.2)	
GC deaths in SDR			
no (Ref)	16	5.5 (1.0-30.8)	
yes	37	14.0 (2.0-50.0)	0.174
Baseline TB			
Negative (Ref)	35	6.0 (1.0-16.0)	
Positive	18	39.0 (15.2-64.5)	<0.001
Overall TB across surveillance			
Negative (Ref)	32	5.5 (1.0-15.5)	
Positive	21	37.0 (9.0-65.0)	<0.001
Baseline RB			
Negative (Ref)	31	6.0 (1.0-13.5)	
Positive	22	42.5 (15.0-65.8)	<0.001
Overall RB across surveillance			
Negative (Ref)	20	5.0 (1.0-10.0)	
Positive	33	22.0 (5.0-61.0)	0.002

SRCC, Signet ring cell carcinoma; PTG, Prophylactic total gastrectomy; TB: targeted biopsy; RB: Random biopsy.

Table S4.

Studies	**Friedman et al 2021[23]**	**Van Dieren et al 2020[16]**	**Asif et al 2023[7]**	**Lee et al 2023[8]**	**Wu et al 2025[10]**
Design	Retrospective	Retrospective	Prospective	Prospective	Prospective
Inclusion criteria	CDH1 mutation carriers	CDH1 mutation carriers	Pathogenic or likely pathogenic CDH1 carriers	Individuals fulfilling HDGC testing criteria	Pathogenic or likely pathogenic CDH1 carriers
Number of patients	48	42	270	145	147
Number of endoscopy	124	98	467	512	541
Number of random biopsies per endoscopy	30	30 (only baseline)	82	24	24
Diagnostic yield of random biopsies	N/A	0.9% (14/5,163)	3.0% (1,150/38,396)	0.7% (86/11,973)	0.8% (106/12,747)
Diagnostic yield of targeted biopsies	N/A	**11% (41/377)**	**3.2% (13/407)**	**8.7% (68/786)**	**14.0% (113/810)**
Percentage of patients with SRCC detected during endoscopy surveillance	N/A	50% (21/42)	63% (76/120)	40% (58/145)	44.9% (66/147)
Percentage of PTG patients with SRCC detected during endoscopy	22% (7/32)	60% (18/30)	57.1% (56/98)	N/A	71.7% (38/53)
Percentage of PTG patients with SRCC detected under PTG	96.9% (31/32)	86.7% (26/30)	96.9% (95/98)	88.9% (32/36)	86.8% (46/53)

HDGC, Hereditary diffuse gastric cancer; SRCC, Signet ring cell carcinoma; PTG, Prophylactic total gastrectomy.

Figure S1

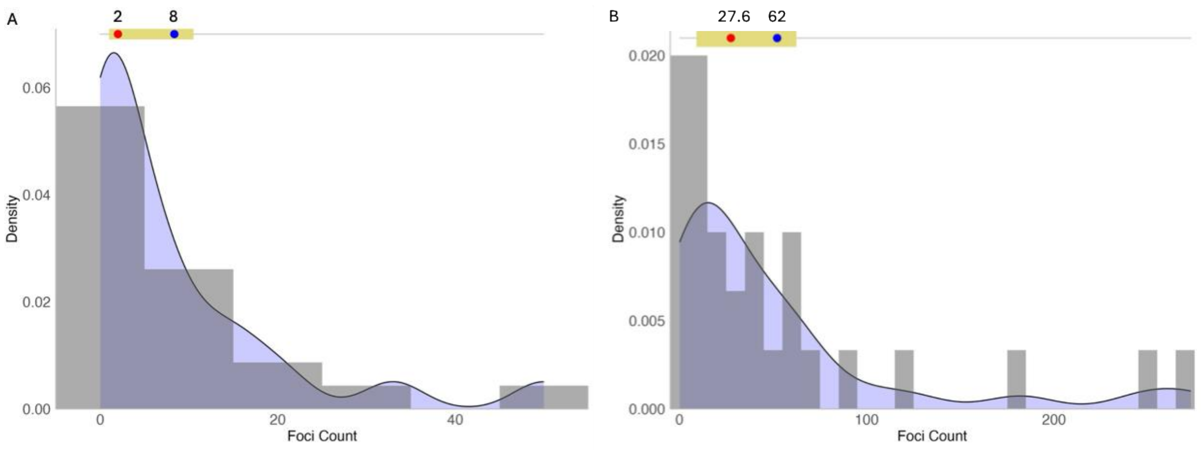
A
2
8
0.06
0.04
0.02
0.00
Density
0
20
40
Foci Count
B
27.6
62
0.020
0.015
0.010
0.005
0.000
Density
0
100
200
Foci Count

Figure S2

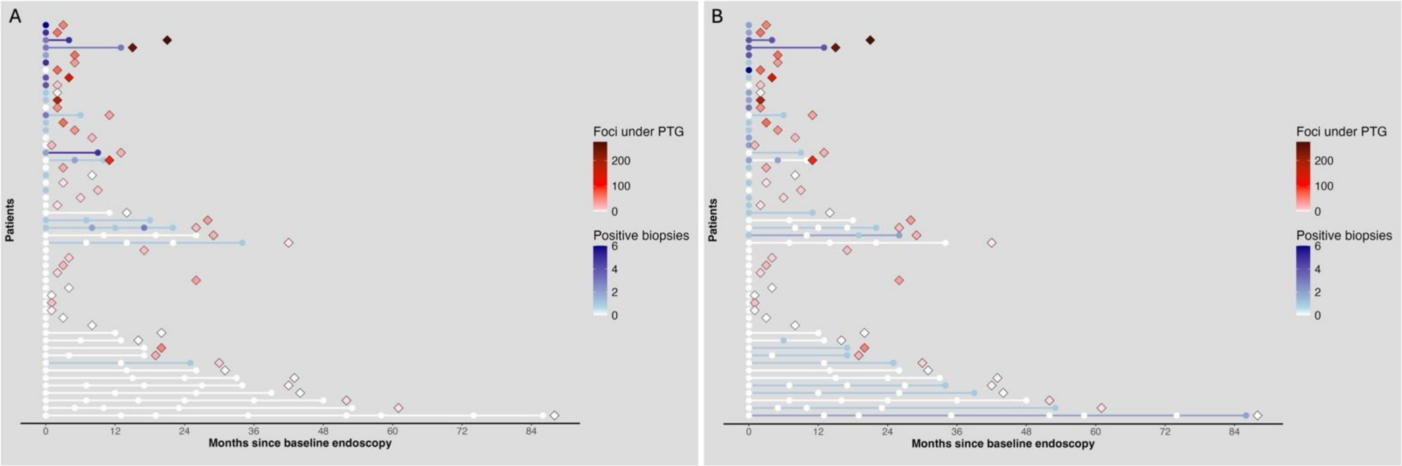
A
Patients
Months since baseline endoscopy
0
12
24
36
48
60
72
84
Foci under PTG
200
100
0
Positive biopsies
6
4
2
0
B
Patients
Months since baseline endoscopy
Foci under PTG
Positive biopsies

Figure S3

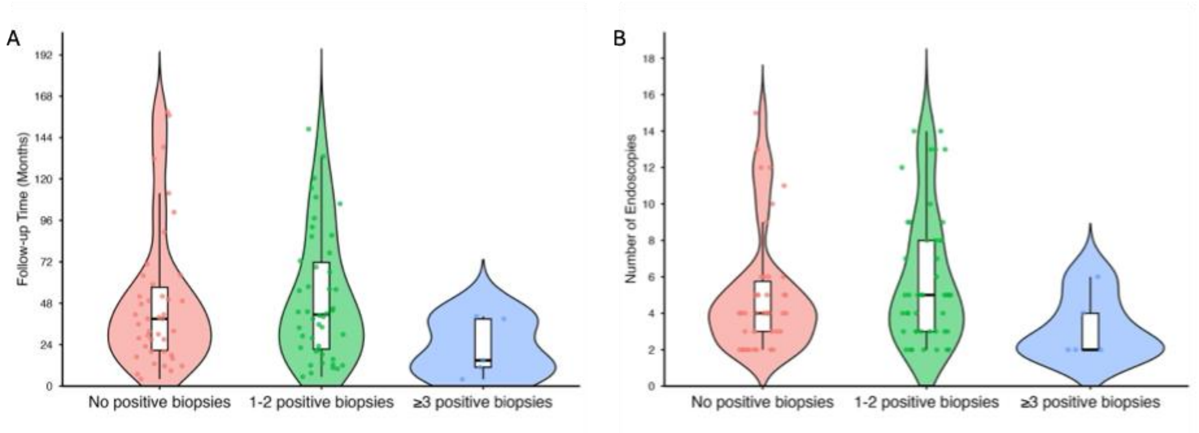
A
Follow-up Time (Months)
0
24
48
72
96
120
144
168
192
No positive biopsies
1-2 positive biopsies
≥3 positive biopsies
B
Number of Endoscopies
0
2
4
6
8
10
12
14
16
18
No positive biopsies
1-2 positive biopsies
≥3 positive biopsies

Figure S4

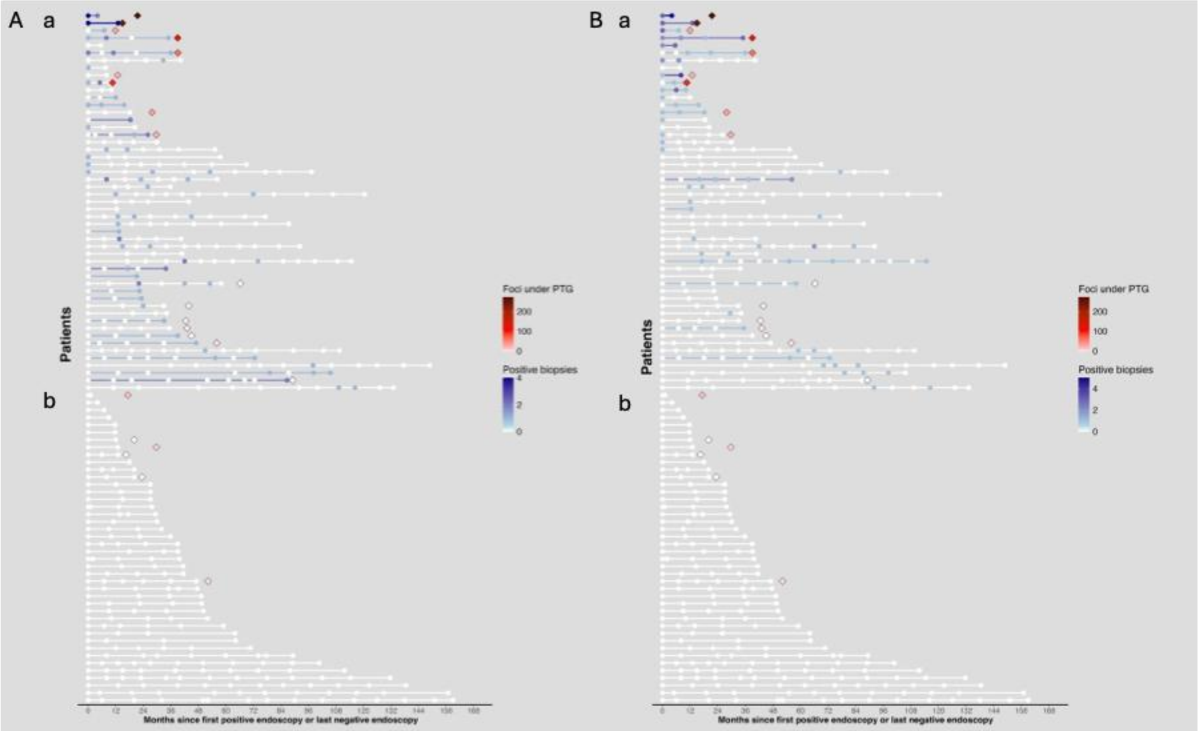
A
a
b
Patients
Foci under PTG
200
100
0
Positive biopsies
4
2
0
0 12 24 36 48 60 72 84 96 108 120 132 144 156 168
Months since first positive endoscopy or last negative endoscopy
B
a
b
Patients
Foci under PTG
200
100
0
Positive biopsies
4
2
0
Months since first positive endoscopy or last negative endoscopy

Figure S5

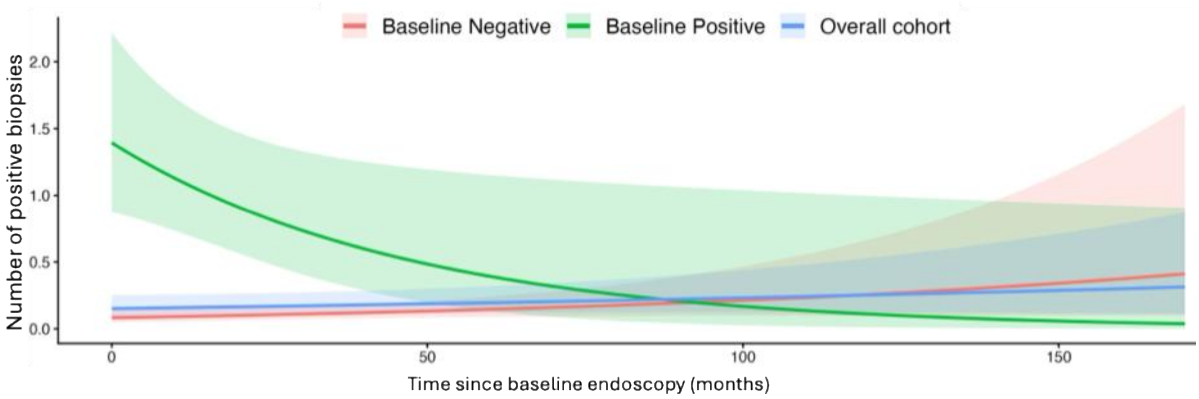
Baseline Negative
Baseline Positive
Overall cohort
Number of positive biopsies
2.0
1.5
1.0
0.5
0.0
0
50
100
150
Time since baseline endoscopy (months)

Figure S6

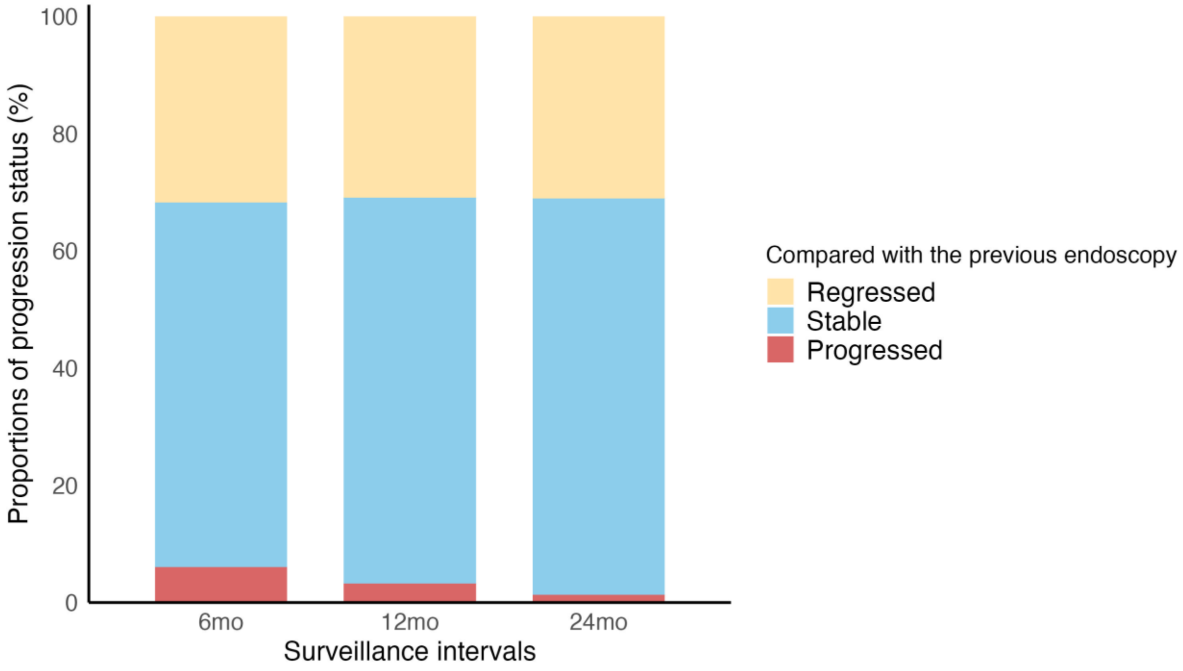
100
80
60
40
20
0
Proportions of progression status (%)
6mo
12mo
24mo
Surveillance intervals
Compared with the previous endoscopy
Regressed
Stable
Progressed
